# Prediction of Impending Type 1 Diabetes through Automated Dual-Label Measurement of Proinsulin:C-Peptide Ratio

**DOI:** 10.1371/journal.pone.0166702

**Published:** 2016-12-01

**Authors:** Annelien Van Dalem, Simke Demeester, Eric V. Balti, Bart Keymeulen, Pieter Gillard, Bruno Lapauw, Christophe De Block, Pascale Abrams, Eric Weber, Ilse Vermeulen, Pieter De Pauw, Daniël Pipeleers, Ilse Weets, Frans K. Gorus, Belgian Diabetes Registry

**Affiliations:** 1 Diabetes Research Center, Brussels Free University—VUB, Brussels, Belgium; 2 Department of Clinical Chemistry and Radio-immunology, Universitair Ziekenhuis Brussel, Brussels, Belgium; 3 Department of Diabetology, Universitair Ziekenhuis Brussel, Brussels, Belgium; 4 Department of Endocrinology, Universitair Ziekenhuis Leuven, Belgium; 5 Department of Endocrinology, Universitair Ziekenhuis Gent, Ghent, Belgium; 6 Department of Endocrinology, Diabetology and Metabolism, Universitair Ziekenhuis Antwerpen, Edegem, Belgium; 7 Department of Endocrinology and Diabetology, GZA Campus Sint Augustinus en Sint Vincentius, Wilrijk-Antwerp, Belgium; 8 Department of Endocrinology and Diabetology, Clinique du Sud Luxembourg et Clinique Saint Joseph, Arlon, Belgium; Jichi Medical University, JAPAN

## Abstract

**Background:**

The hyperglycemic clamp test, the gold standard of beta cell function, predicts impending type 1 diabetes in islet autoantibody-positive individuals, but the latter may benefit from less invasive function tests such as the proinsulin:C-peptide ratio (PI:C). The present study aims to optimize precision of PI:C measurements by automating a dual-label trefoil-type time-resolved fluorescence immunoassay (TT-TRFIA), and to compare its diagnostic performance for predicting type 1 diabetes with that of clamp-derived C-peptide release.

**Methods:**

Between-day imprecision (n = 20) and split-sample analysis (n = 95) were used to compare TT-TRFIA (AutoDelfia, Perkin-Elmer) with separate methods for proinsulin (in-house TRFIA) and C-peptide (Elecsys, Roche). High-risk multiple autoantibody-positive first-degree relatives (n = 49; age 5–39) were tested for fasting PI:C, HOMA2-IR and hyperglycemic clamp and followed for 20–57 months (interquartile range).

**Results:**

TT-TRFIA values for proinsulin, C-peptide and PI:C correlated significantly (r^2^ = 0.96–0.99; *P*<0.001) with results obtained with separate methods. TT-TRFIA achieved better between-day %CV for PI:C at three different levels (4.5–7.1 vs 6.7–9.5 for separate methods). In high-risk relatives fasting PI:C was significantly and inversely correlated (r_s_ = -0.596; *P*<0.001) with first-phase C-peptide release during clamp (also with second phase release, only available for age 12–39 years; n = 31), but only after normalization for HOMA2-IR. In ROC- and Cox regression analysis, HOMA2-IR-corrected PI:C predicted 2-year progression to diabetes equally well as clamp-derived C-peptide release.

**Conclusions:**

The reproducibility of PI:C benefits from the automated simultaneous determination of both hormones. HOMA2-IR-corrected PI:C may serve as a minimally invasive alternative to the more tedious hyperglycemic clamp test.

## Introduction

There is growing consensus that immune interventions in type 1 diabetes should concentrate on the presymptomatic disease phase [1–3]. Development of multiple islet autoantibodies indicates a point of no return towards development of type 1 diabetes within 20 years in individuals at familial or *HLA-DQ*-inferred risk but less than 20% of them progress within 2–3 years [3–5]. Immune intervention trials in asymptomatic type 1 diabetes require identification of a subgroup with a much higher overall progression rate in the short term, in order to reach conclusions within a reasonable timeframe [5, 6]. We recently reported that detection of a decreased first- or second phase C-peptide release during hyperglycemic clamp–the gold standard for beta cell function assessment [7, 8]–could serve this purpose, particularly when applied in individuals positive for autoantibodies directed against IA-2 (IA-2A) or zinc transporter 8 (ZnT8A) [9–12].

For large scale implementation more simple, minimally invasive alternatives to the hyperglycemic clamp are warranted [5, 13]. An increased proinsulin:C-peptide ratio (PI:C) has been proposed as functional screening marker to identify multiple autoantibody-positive individuals at high risk of impending diabetes [14–16]. However, nutrient intake and the additive errors of the proinsulin (PI) and C-peptide assays may negatively influence consistency of PI:C during follow-up [14, 17]. When using separate immunoassays, between-assay imprecision of this ratio ranged between 9.0 and 11.8% [14, 17]. This variability could be improved by selecting precise methods to determine both hormones separately [18, 19] or by measuring both peptides simultaneously in the same reaction vessel [17]. We therefore developed a trefoil-type time-resolved fluorescence immunoassay (TT-TRFIA) for simultaneous measurement of C-peptide and PI, taking advantage of a common monoclonal capture antibody against the C-terminus of C-peptide, and two differentially labeled monoclonal detection antibodies: one directed against the N-terminus of C-peptide, and another against an epitope of the insulin moiety of PI [17].

Here we have further automated this test by adapting it to the Autodelfia 1235 instrument (Perkin-Elmer, Massachusetts, USA) which is likely to further reduce assay imprecision and is also warranted in view of the screening effort needed to identify individuals with impending diabetes among family members and, even more so, in the general population [20]. In the present report we compared the analytical performance of this automated TT-TRFIA for PI:C with that of state-of-the-art assays for C-peptide [18] and PI [19]. We next investigated the diagnostic performance of the automated dual-label TT-TRFIA to predict progression to diabetes within 2 years in first-degree relatives at high autoantibody-inferred risk in parallel with that of clamp-derived C-peptide release.

## Methods

### Participants

Offspring and siblings (n = 49; 5–39 years) of type 1 diabetes patients at high autoantibody-inferred risk (IA-2A^+^ or ZnT8A^+^ plus ≥1 other autoantibody; ca 45% 5-year risk) [9] were enrolled by the Belgian Diabetes Registry and underwent a metabolic assessment, consisting of an oral glucose tolerance test (OGTT) and a hyperglycemic clamp test and were followed every 6 to 12 months. Children (n = 13; 5–11 years) only underwent the first 10 min of hyperglycemia [12]. Baseline samples of the clamp were used to assess fasting PI:C, PI, C-peptide, glucose and HOMA2-IR. Progression to diabetes was ascertained as before [12]. At diagnosis according to American Diabetes Association criteria [21] patients were shifted to intensive insulin treatment.

Written informed consent was obtained from each participant or from their parents in case of minors. The study protocol was approved by the Ethics Committees of the Belgian Diabetes Registry and participating university hospitals where the metabolic tests were performed (leading Ethics Committee: Universitair Ziekenhuis Brussel; non-leading Ethics Committees: Universitair Ziekenhuis Antwerpen, Universitair Ziekenhuis Gent, Universitair Ziekenhuis Leuven; B.U.N. 143201422342) and conducted according to the Declaration of Helsinki as revised in 2013 (http://www.wma.net/en/30publications/10policies/b3/, accessed on July 13^th^, 2016).

Hormonal assays were compared by split-sample analysis of anonymous surplus plasma samples from the biobank of the Belgian Diabetes Registry obtained after informed consent (B.U.N. 143201524128) for measuring pancreatic hormones and other early markers of type 1 diabetes at the Department of Clinical Chemistry and Radio-immunology (Universitair Ziekenhuis Brussel) which acts as reference laboratory for the Belgian Diabetes Registry [17].

### Beta cell stimulation tests

During OGTT blood was sampled to determine glucose, PI, C-peptide and PI:C before and at min 15 (only in some relatives), 30, 60, 90 and 120 after an oral glucose load of 1.75 g/kg without exceeding the maximum of 75 g [11, 12]. A hyperglycemic clamp test was performed 1–2 weeks later, as previously described [12]. Briefly, after an overnight fast, 1.1 mol/L glucose (Baxter, Brussels, Belgium) was infused via the left antecubital vein at time 0 and blood for hormone measurements was drawn from the contralateral vein. The blood glucose level was raised to reach a plateau of 10 mmol/L. After a priming dose of glucose, the hyperglycemic target was maintained by adjusting the glucose infusion rate upon assessment of bedside blood glucose levels every 5 min (Accu-chek® Inform II, Roche Diagnostics, Mannheim, Germany) [12]. Blood samples were collected before and at min 2.5, 5, 7.5, 10, 120, 135 and 150 after start. The trapezoidal rule was used to calculate area under the curve (AUC) of C-peptide release between min 5–10 (AUC_5-10 min_ C-peptide) and min 120–150 (AUC_120-150min_ C-peptide) [11]. In healthy controls, the intra-individual variability of AUC_5-10min_ C-peptide and AUC_120-150min_ C-peptide amounted to 11.8% and 11.7% respectively, hereby outperforming reported data for intravenous glucose tolerance tests [11].

Venous whole blood was collected in NaF tubes for glucose and K-EDTA tubes (Sarstedt, Nümbrecht,Germany) containing aprotinin (Trasylol; Bayer, Brussels, Belgium); final concentration 600 kallikrein inactivator units/mL) on ice for hormonal assays and glycated hemoglobin (HbA1c). After centrifugation at 1600g for 15min, plasmata were aliquoted and stored at -80°C. Under these conditions, hormone levels were stable long-term ([22] and own unpublished data).

### Analytical methods

Insulin autoantibodies (IAA), GAD65 autoantibodies (GADA), IA-2A and ZnT8A were determined in serum by liquid phase radiobinding assays (8, 10). Glucose was measured on Vitros (O-CD, Rochester, NY) and HbA1c by HPLC (Tosoh, Tokyo, Japan).

PI:C was determined by TT-TRFIA which allows simultaneous measurement of C-peptide and PI (S1 Table). Briefly, the common monoclonal capture antibody (mAb PEP-001, DAKO, Glostrup, Denmark) has epitope specificity for C-terminal C-peptide. All captured molecules containing the intact C-peptide sequence are detected by a second Eu^3+^-labelled (Perkin-Elmer) mAb CPT-3F11 (DAKO) directed against N-terminal C-peptide, and captured molecules containing an insulin moiety (proinsulin and conversion intermediates) [17] by a third in-house biotinylated mAb (HUI 001; gift from Dr. Pass, Novo-Nordisk, Bagsvaerd, Denmark) [23] in combination with Tb^3+^-labeled streptavidin (Perkin-Elmer). The use of two different fluorescent labels allows to quantify the two detecting antibodies within the same reaction compartment and the difference between both signals is a measure of the amount of true C-peptide. For this study, the assay was adapted to the Autodelfia 1235 automated instrument (Perkin-Elmer) and an outprint of the parameters for this application is shown in S1 File. Due to the high cross-reactivity with conversion intermediates, the PI assay measures total PI immunoreactive material [14], which is not a disadvantage as conversion intermediates are also reported to increase in prediabetes [24, 25]. C-peptide levels up to at least 7000 pmol/L did not interfere with PI measurements [18]. Because of the 100% cross-reactivity of PI in the C-peptide assay, free C-peptide levels were obtained by subtracting the PI concentration from the total C-peptide result [17].

For method comparison by split-sample analysis, C-peptide and PI were also measured separately by automated state-of-the-art methods, using an electrochemiluminescence immunoassay (ECLIA; Cobas e411/Elecsys, Roche C-peptide kit) [18] and an in-house TRFIA (Autodelfia, Perkin-Elmer) [19] respectively. Between-day imprecision was determined by analyzing three pooled human plasma samples containing different levels of the analytes in duplicate during 20 different runs on 20 different days [26].

### Statistics

Statistical analyses were performed two-tailed using SPSS version 22.0 for Windows (IBM SPSS Statistics, Chicago, IL, USA) and considered significant if *P*<0.05 or <0.05/k for k comparisons (Bonferroni adjustment). Figures were generated with GraphPad Prism version 5.00 for Windows (San Diego, CA, USA). Differences between groups were assessed by Mann-Whitney U or Kruskall-Wallis tests for continuous variables and by χ^2^ test or Fisher’s exact test for categorical variables. Deming regression was used for method comparisons and Spearman-rank correlation coefficient (r_s_) to assess correlations between variables. Prediction of 2-year progression to diabetes in high-risk relatives was assessed by ROC analysis comprising calculation of the AUC (95% CI) under the ROC curve (AUC-ROC), diagnostic sensitivity, specificity and accuracy and the Akaike Information Criterion (AIC). AUC-ROCs were compared according to [27] and variances according to [28]. HOMA2-IR was calculated with https://www.dtu.ox.ac.uk/homacalculator/. Independent predictors of diabetes onset were assessed using two-by-two Cox regression analysis to comply with Vittinghoff’s criterion [29]. Most relatives (92%) who did not develop diabetes during the study completed 2-year follow-up.

## Results

### Analytical performance of the automated TT-TRFIA

Over a wide concentration range (100–4400 pmol/L C-peptide; 1.5–190 pmol/L PI), C-peptide, PI and PI:C plasma levels obtained by automated TT-TRFIA were significantly correlated (r^2^ = 0.96–0.99; *P*<0.001) with those from separate methods (TRFIA for PI and ECLIA for C-peptide) (S1 Fig). The 95% CI for slope and intercept of the regression line were respectively 1.01–1.05 and -61.9–2.81 pmol/L for C-peptide, 1.14–1.20 and -0.86–1.44 pmol/L for PI, and 0.92–1.00 and 0.2–0.51% for PI:C.

Despite the fact that ECLIA achieved lowest CVs for C-peptide (2.2–2.8%), TT-TRFIA could determine PI:C with greater precision (4.5–7.1%CV) than the combination of two separate methods (TRFIA and ECLIA; 6.7–9.5%), especially for PI:C>2% (Table 1). At the highest PI:C level, the %CV for TT-TRFIA was significantly lower than obtained with the two separate assays (*P*<0.001 by Levene test) [28].

**Table 1:** Between-day imprecision (n = 20) of C-peptide, PI and PI:C, determined separately with two automated methods (ECLIA and TRFIA) or simultaneously with the automated TT-TRFIA in pooled human plasma.

		Low level		Intermediate level		High level	
Analyte	Method	Mean ± SD	%CV	Mean ± SD	%CV	Mean ± SD	%CV
**C-peptide**	ECLIA	358 ± 10 pmol/L	2.8	500 ± 14 pmol/L	2.8	1537 ± 35 pmol/L	2.3
	TT-TRFIA	327 ± 25 pmol/L	7.5	469 ± 34 pmol/L	7.2	1616 ± 83 pmol/L	5.1
**PI**	TRFIA	4.2 ± 0.3 pmol/L	7.5	14.4 ± 0.9 pmol/L	6.4	73.7 ± 6.4 pmol/L	8.7
	TT-TRFIA	4.8 ± 0.3 pmol/L	6.9	14.4 ± 1.2 pmol/L	8.1	77.4 ± 4.4 pmol/L	5.7
**PI:C**	TRFIA/ECLIA	1.18 ± 0.09%	7.9	2.97 ± 0.20%	6.7	5.04 ± 0.48%	9.5
	TT-TRFIA	1.49 ± 0.11%	7.1	3.18 ± 0.17%	5.2	5.03 ± 0.23%	4.5

Between-method comparison of mean PI:C ratios (Table 1) revealed slightly higher values for TT-TRFIA than with separate assays, the difference decreasing with increasing ratios. This is compatible with the tendency towards lower C-peptide and higher PI values measured with TT-TRFIA in the lower concentration ranges. This difference becomes relatively less important at higher hormone levels as also indicated by the regression equations in S1 Fig.

### Diagnostic performance of TT-TRFIA for impending diabetes

**Fig 1:**
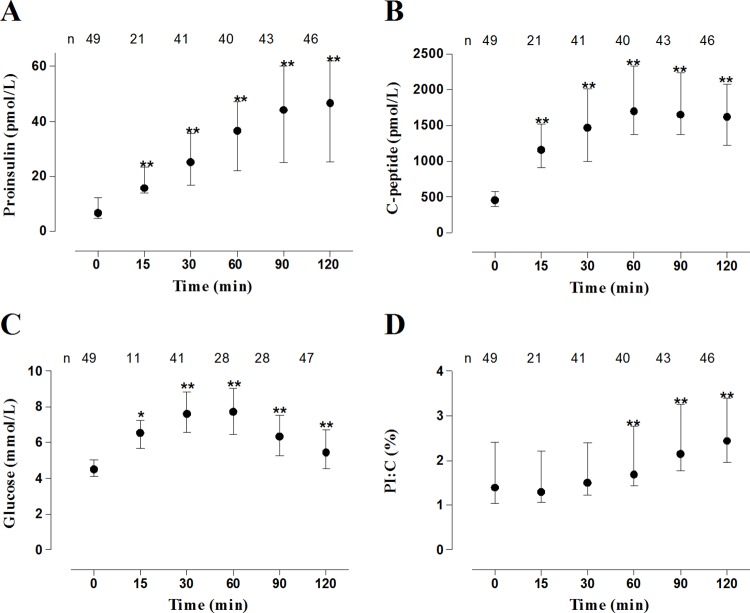
Evolution of proinsulin (A), C-peptide (B), glucose (C) and PI:C ratio (D) during OGTT in relatives at high autoantibody-inferred risk; *P = 0.003; **P<0.001. The number of available samples tested is indicated above each time point.

The PI:C ratio was found to increase to a variable degree with time after glucose load during OGTT (Fig 1) without amplifying the differences between those who progressed to diabetes during follow-up and those who did not (not shown). In search for functional markers that could be determined on a single blood sample, we decided to use fasting samples instead of random samples to evaluate the potential of PI:C ratio for prediction of impending diabetes to optimize consistency of results. These samples also allow to calculate HOMA2-IR. A longitudinal pilot study of PI:C values in autoantibody-positive relatives who rapidly progressed to diabetes from baseline clamp indicated an increasing trend in PI:C within 2 years before diagnosis, with decreasing HOMA2-IR in many relatives: consequently the ratio of PI:C over HOMA2-IR tended to increase even steeper before diagnosis (S2 Fig). In the present study, HOMA2-IR was significantly and inversely correlated with clamp-derived insulin sensitivity index (ISI, r_s_ = -0.712; *P* = 0.001; only available in participants aged 12–39 years who underwent a full clamp of 150 min) [11], hereby validating its use in the investigated cohort. Based on these observations, we compared the capacity of PI:C–with or without adjustment for HOMA2-IR–with that of clamp-derived AUC C-peptide to predict 2-year progression to diabetes.

**Fig 2:**
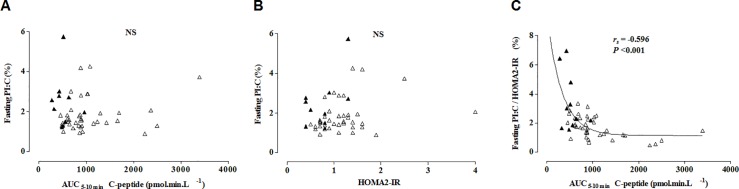
Relation between fasting PI:C and AUC_5-10min_ C-peptide (A), fasting PI:C and HOMA2-IR (B) and fasting PI:C corrected for HOMA2-IR and AUC_5-10min_ C-peptide (C) in relatives at high autoantibody-inferred risk (HR) (IA-2A^+^ or ZnT8A^+^ plus ≥ 1 other autoantibody) (9). Filled triangles = progressors within 2 years (n = 10), open triangles = slow-/non-progressors (n = 39). AUC_5-10min_ C-peptide: first-phase AUC C-peptide release during hyperglycemic clamp test (min 5–10); HOMA2-IR: homeostatic model assessment for insulin resistance; r_s_: Spearman's rank correlation coefficient; NS: not significant

During a median (interquartile range) follow-up of 36 (20–57) months, 25 of 49 relatives at high autoantibody-inferred risk developed diabetes, 10 of whom within 2 years from baseline clamp. Relatives who did not yet progress to diabetes (n = 24) and those who did progress after more than 2 years from baseline clamp (n = 15) did not differ significantly in baseline characteristics and were considered together (not shown). In comparison with both groups combined (n = 39) rapid progressors from baseline clamp tended to have lower values for clamp-derived AUC_5-10min_ C-peptide, fasting C-peptide and HOMA2-IR, and higher values for HbA1c and fasting PI:C (especially when corrected for HOMA2-IR) (Table 2). Both groups did not differ in prevalence of *HLA-DQ* genotypes or the various autoantibody types (Table 2), nor in autoantibody levels, regardless of whether all relatives were considered or only those positive for a particular autoantibody specificity (not shown). Fasting PI:C was not correlated with AUC_5-10min_ C-peptide (Fig 2A) or HOMA2-IR (Fig 2B). However, normalizing PI:C for HOMA2-IR unveiled a highly significant hyperbolic correlation (r_s_ = -0.596; *P*<0.001) with AUC_5-10min_ C-peptide, andalso with AUC_120-150min_ C-peptide, only available for age 12–39 years (n = 31; r_s_ = -0.529; *P* = 0.002) (Fig 2C). Both in healthy controls (n = 59) and in relatives with the high-risk autoantibody profile PI:C was significantly correlated with body mass index (BMI) z-score (r_s_ = 0.417; *P* = 0.001 and r_s_ = 0.357; *P* = 0.015, respectively; data available for 46 relatives), was similar in males and females, but was higher in individuals under age 20 years than in those aged 20 years or more. In contrast, HOMA2-IR conducted PI:C was independent of BMI z-score, sex and age (data not shown).

**Table 2:** Characteristics of relatives at high autoantibody-inferred risk (HR) (IA-2A^+^ or ZnT8A^+^ plus ≥ 1 other autoantibody) [9] according to progression rate to diabetes.

		Progressors to diabetes		
Characteristics		Within 2 years	After 2 years/not yet	*P*
**Baseline**				
n		10	39	
Age, years		15 (8–24)	16 (12–23)	0.487
Sex, n males/n females		6/4	21/18	1.000
BMI^a^, z-score		-0.33 (-1.13–1.54)	0.20 (-0.56–1.53)	0.252
HbA1c, %		5.6 (5.4–5.7)	5.3 (5.1–5.4)	**0.015**
Fasting glucose, mmol/L		4.4 (3.9–5.3)	4.3 (4.1–4.7)	0.628
Fasting proinsulin, pmol/L		5.8 (4.1–13.9)	8.5 (5.2–12.1)	0.348
Fasting C-peptide, pmol/L		385 (188–454)	537 (392–691)	**0.010**
Fasting PI:C ratio, %		2.37 (1.46–2.84)	1.57 (1.32–1.95)	0.080
Fasting PI:C ratio / HOMA2-IR^b^, %		2.66 (1.82–5.21)	1.64 (1.18–2.35)	**0.005**
AUC_5-10min_ C-peptide^c^, pmol.min.L^-1^		494 (396–576)	877 (678–1152)	**< 0.001**
AUC_5-10min_ C-peptide^c^ / HOMA2-IR^b^, pmol.min.L^-1^		664 (484–1092)	892 (722–1111)	0.107
HOMA2-IR^b^		0.8 (0.4–1.0)	1.1 (0.8–1.4)	**0.021**
Antibody seropositivity^a^^,^^d^				
	IAA, n (%)	6 (60)	18 (47)	0.477
	GADA, n (%)	9 (90)	35 (92)	1.000
	IA-2A, n (%)	9 (90)	32 (84)	1.000
	ZnT8A, n (%)	9 (90)	36 (95)	0.512
	Number of autoantibodies	4 (2–4)	3 (3–4)	0.490
*HLA-DQ* haplotype				
	*DQ2*/*DQ8*, n (%)	4 (40)	9 (23)	0.422
	*DQ8*/non*DQ2*, n (%)	3 (30)	20 (54)	0.299
	*DQ2*/non*DQ8*, n (%)	2 (20)	7 (18)	1.000
	non*DQ2*/non*DQ8*, n (%)	1 (10)	3 (8)	1.000
**Follow-up from baseline**, months		8 (5–19)	39 (34–60)	**< 0.001**

Parameters measured during hyperglycemic clamp test unless otherwise indicated; data are median, n (n/n) or n (%)

^a^measured at the time of OGTT

^b^HOMA2-IR, homeostatic model assessment for insulin resistance

^c^AUC_5-10min_, first-phase AUC C-peptide release during hyperglycemic clamp test (min 5–10); threshold for significance: *P*<0.05/22 or *P*<0.0023 (Bonferroni correction)

^d^no significant difference between both groups in IAA, GADA, IA-2A or ZnT8A levels in IAA^+^, GADA^+^, IA-2A^+^ or ZnT8A^+^ relatives, respectively

All variables from Table 2 were tested in univariate Cox regression for prediction of 2-year progression to diabetes and those with *P*<0.1 were entered in two-by-two multivariate models against PI:C without (Model 1) or with (Model 2) normalization for HOMA2-IR (Table 3). Fasting PI:C remained an independent predictor of impending diabetes (*P* = 0.035–0.001) together with AUC_5-10min_ C-peptide (*P*<0.001), fasting C-peptide (*P* = 0.001), HOMA2-IR (*P* = 0.001) and HbA1c (*P* = 0.044), respectively, whereas PI:C/HOMA2-IR outperformed all other parameters (Table 3), and the most informative OGTT-derived parameters [12] as well (not shown). In high-risk relatives with normal glucose tolerance at baseline (n = 44) only AUC_5-10min_ C-peptide or HOMA2-IR- adjusted PI:C predicted diabetes onset within 2 years (7 events; not shown). ROC-curve analysis for fasting blood glucose and for the parameters associated with 2-year progression to type 1 diabetes in Table 3 showed that PI:C/HOMA2-IR achieved values for AUC under the ROC-curve (AUC-ROC), diagnostic accuracy, and AIC that came closest to the values observed for AUC_5-10min_ C-peptide (Table 4; not significantly different according to [27]). Combinations of AUC_5-10min_ or HOMA-corrected PI:C with 2 or more markers derived from fasting samples tended to improve their AUC-ROC values without reaching significance (only a selection of all possible combinations are shown in Table 4, including those with the highest values). Using criteria for cutoff values defined in previous publications [11, 12, 14], AUC_5-10min_ C-peptide (< percentile 10 of healthy controls) and HOMA2-IR-adjusted PI:C (≥ percentile 66 of healthy controls) both identified 7 of 10 relatives who progressed within 2 years.

**Table 3:** Cox regression analysis for 2-year progression (10 events) to type 1 diabetes in 49 first-degree relatives at high autoantibody-inferred risk (HR) (IA-2A^+^ or ZnT8A^+^ plus ≥ 1 other autoantibody) [9]. All variables from Table 2 were tested univariately. Only those with univariate *P*<0.1 are shown here and were entered in two-by-two multivariate models.

	Univariate	Multivariate		Multivariate		Multivariate		Multivariate	
Independent variable	*P*	*P*	HR 95% CI^a^	*P*	HR 95% CI^a^	*P*	HR 95% CI^a^	*P*	HR 95% CI^a^
**Model 1**									
Fasting PI:C	**0.024**	**0.008**	1.27–4.96	**0.001**	1.77–8.52	**0.001**	1.80–8.61	**0.035**	1.05–3.41
AUC_5-10min_ C-peptide^b^	**0.001**	**< 0.001**	0.990–0.997	-	-	-	-	-	-
Fasting C-peptide	**0.013**	-	-	**0.001**	0.987–0.997				
HOMA2-IR^c^	**0.022**	-	-	**-**	-	**0.001**	0.002–0.185	-	-
HbA1c	**0.035**	**-**	-	-	-	-	-	**0.044**	1.07–89.5
**Model 2**									
Fasting PI:C / HOMA2-IR^c^	**< 0.001**	**< 0.001**	1.35–2.52	**< 0.001**	1.42–2.76	**< 0.001**	1.35–2.52	**< 0.001**	1.35–2.50
AUC_5-10min_ C-peptide^b^	**0.001**	0.077	-	-	-	-	-	-	-
Fasting C-peptide	**0.013**	-	-	0.469	-	-	-	-	-
HOMA2-IR^c^	**0.022**	-	-	-	-	0.456	-	-	-
HbA1c	**0.035**	**-**	-	-	-	-	-	0.079	-

^a^95% confidence interval of hazard ratio

^b^AUC_5-10min_, first-phase AUC C-peptide release during hyperglycemic clamp test (min 5–10)

^c^HOMA2-IR, homeostatic model assessment for insulin resistance; threshold for significance for multiple two-by-two multivariate analyses: *P*<0.05/8 or *P*<0.0063

**Table 4:** Receiver operating characteristic (ROC) analysis for prediction of progression to diabetes within 2 years in high autoantibody-inferred risk (HR) (IA-2A^+^ or ZnT8A^+^ plus ≥ 1 other autoantibody) [9].

			ROC-AUC^a^	95% CI	Sensitivity (%)	Specificity (%)	Accuracy (%)	AIC^b^
Fasting glucose			0.55	0.31–0.79	40	90	80	
Fasting PI:C			0.68	0.49–0.87	70	77	74	50.2
HOMA2-IR^c^			0.74	0.56–0.92	80	59	67	46.3
HbA1c			0.75	0.57–0.94	70	82	79	48.7
Fasting C-peptide			0.78	0.63–0.93	100	49	56	41.5
Fasting PI:C / HOMA2-IR^c^			0.79	0.64–0.94	50	95	84	40.4
AUC_5-10min_ C-peptide^d^			0.88	0.75–1.00	90	82	84	34.8
AUC_5-10min_ C-peptide^d^								
	+ Fasting glucose		0.89	0.76–1.00	90	87	88	36.4
	+ Fasting PI:C		0.90	0.77–1.00	90	90	90	33.5
	+ HOMA2-IR^c^		0.89	0.76–1.00	90	87	88	36.8
	+ HbA1c		0.91	0.80–1.00	90	84	85	32.8
	+ Fasting C-peptide		0.87	0.72–1.00	89	82	83	36.5
	+ Fasting PI:C / HOMA2-IR^c^		0.92	0.80–1.00	90	87	88	34.2
Fasting PI:C / HOMA2-IR^c^								
	+ Fasting glucose		0.82	0.68–0.96	90	62	67	41.3
	+ HbA1c		0.82	0.63–1.00	80	79	79	40.2
	+ Fasting C-peptide		0.85	0.72–0.97	100	59	67	36.7
	+ HOMA2-IR + HbA1c + fasting glucose		0.93	0.85–1.00	100	82	85	36.2

^a^AUC under the ROC-curve

^b^Akaike Information Criterion

^c^HOMA2-IR, homeostatic model assessment for insulin resistance

^d^AUC_5-10min_, first-phase AUC C-peptide release during hyperglycemic clamp test (min 5–10)

Of note, 2h post-glucose load C-peptide over glucose ratio–recently proposed as a marker of beta cell function [30]–performed equally well as HOMA2-IR corrected PI:C in ROC analysis (AUC-ROC = 0.81 (95% CI 0.66–0.96); NS vs PI:C/HOMA2-IR; not shown), but was not further considered here because we focused on biomarkers that could be determined on fasting samples. In the present study the AUC-ROC of PI:C obtained with separate methods for PI and C-peptide was not significantly worse than that achieved by TT-TRFIA (not shown).

## Discussion

The automated TT-TRFIA for simultaneous measurement of PI, C-peptide and their ratio was shown to generate results that correlate well with values obtained with singleplex state-of-the-art methods for both hormones, while achieving better precision for PI:C. Using this new assay format, fasting PI:C significantly and inversely correlated with clamp-derived AUC C-peptide in relatives at high autoantibody-inferred risk, but only after normalization for HOMA2-IR, and predicted impending diabetes equally well as the gold standard [7, 8].

Reproducibility of PI:C benefitted from assay automation and the lack of additive effects of both analytes’ imprecision [17]: indeed, TT-TRFIA tended to outperform both the combination PI-TRFIA/C-peptide ECLIA and reported values for the manually performed TT-TRFIA (6.4–11% CV) in this respect [17]. Better precision is likely to improve consistency of results during follow-up, but whether it also translates into a higher predictive value of the ratio determined with TT-TRFIA remains to be investigated in larger studies. The % CVs obtained with C-peptide ECLIA and PI TRFIA are in line with published values [18, 19, 31, 32], but it is evident that the use of separate methods with smaller CV’s (especially for PI) might have a further beneficial effect on the predictive value of the PI:C ratio.

Other strengths of the study include the use of 2-year progression to diagnosis, which is a key instrument for secondary prevention trials with immune intervention [5], and the use of fasting plasma samples which is likely to optimize consistency of PI:C values during follow-up [14] and allows calculation of HOMA2-IR. Our preliminary data are limited by the number of fast progressors from baseline clamp, and should be confirmed in larger, longitudinal studies in independent risk groups. They should also further investigate the exact relationship between changes in PI:C and HOMA2-IR according to time from diabetes onset. As in previous studies [11, 12], we did not use the heated-hand technique in the clamp tests as this is cumbersome for multiple sampling and omitting it was not reported to induce spurious results [33]. First-phase C-peptide release was calculated as AUC_5-10min_ C-peptide which translated in a robust intraindividual reproducibility which equaled that of second-phase release (<12% intraindividual variation) [11].

Our results suggest that PI:C reflects a combination of beta cell function and insulin action as it correlates with BMI z-score, and–after adjustment for HOMA2-IR–with clamp-derived C-peptide release. A disproportionately high PI value, expressed as PI:C or PI:insulin ratio, was proposed to mainly indicate sub(clinical) beta cell dysfunction in (impending) type 2 or type 1 diabetes [15, 34–36]. However, the fact that PI:C only significantly and inversely correlated with clamp-derived AUC C-peptide release when corrected for HOMA2-IR suggests that PI:C also reflects to some degree the level of insulin resistance. In the present group of autoantibody-positive relatives, most individuals had normal to increased insulin sensitivity (HOMA2-IR<1.0; Table 2) but in some the rise in PI or PI:C relative to AUC C-peptide may have been disproportionate due to insulin resistance (see e.g. the point to the right in Fig 2A). Omission of this outlier did not change the (lack of) significance in the various panels of Fig 2. This non-progressor had an elevated PI:C ratio and BMI z-score (2.65), but a low HOMA2-IR adjusted PI:C ratio. Normalization of the PI:C ratio for HOMA2-IR improved its overall correlation with AUC C-peptide (Fig 2C). It also rendered this parameter independent of age, sex and BMI z-score at variance with uncorrected PI:C according to this study and [37].

HOMA2-IR-corrected PI:C may serve as a minimally invasive alternative to the more tedious hyperglycemic clamp test. It outperformed clamp-derived AUC C-peptide in Cox regression analysis, but achieved a slightly–though not significantly–lower AUC-ROC. It requires only a fasting blood sample and is thus more widely applicable than stimulation tests in the age category 5–39 years, the target population for secondary prevention trials consisting of school children and active young adults. In multivariate Cox regression this variable outperformed fasting C-peptide and HbA1c, previously shown to predict time to diabetes in children at risk [38]. AUC-ROC and AIC were slightly though not significantly better for HOMA2-IR corrected PI:C than for HbA1c or fasting C-peptide [27]. Combination of several markers that can be determined on a single fasting blood sample (PI:C, HOMA2-IR, HbA1c, fasting glucose) could slightly, though as yet not significantly, improve the diagnostic performance in ROC-analysis. Larger studies in multiple autoantibody-positive individuals should decide whether a combination of those markers can be moulded into a composite risk score with further improved predictive value. Conclusions remained valid in absence of dysglycemia and were independent of IAA and IA-2A levels (not shown)–reportedly associated with rapid progression in children [39, 40]; indeed, relatives who did progress to diabetes within 2 years from baseline and those who did progress later or not yet, did not differ in antibody levels in our cohort, probably due to our definition of high-risk autoantibody profile (IA-2A^+^ or ZnT8A^+^ plus ≥1 other autoantibody^+^) [9].

Taken together our results indicate that rapid progression to type 1 diabetes associates with a more pronounced deterioration of beta cell function relative to insulin resistance as assessed by HOMA2-IR. They are in line with previous reports that insulin resistance makes only a borderline contribution to risk of progression to diabetes in individuals with an already compromised beta cell function [41–43]. Larger follow-up studies in risk groups comparing HOMA2-IR-corrected PI:C, clamp-derived measures [5], HbA1c [38], and glycemic variability [13] are needed to further document consistency of results over time, and to determine generally applicable cutoff values for HOMA2-IR-corrected PI:C in the perspective of further refining staging of presymptomatic type 1 diabetes [4], identifying rapid progressors and establishing minimally invasive criteria for inclusion in secondary prevention trials.

## Supporting Information

S1 Fig Method comparison for (A) PI (automated TT-TRFIA vs. TRFIA), (B) C-peptide (automated TT-TRFIA vs. ECLIA) and (C) PI/C (automated TT-TRFIA vs. TRFIA/ECLIA) on 95 plasma samples from patients with type 1 diabetes and their first-degree relatives of a T1D patient.
(DOCX)

S2 Fig Consecutive changes in PI:C (A), HOMA2-IR (B) and HOMA2-IR-adjusted PI:C (C) 2 years prior to clinical onset in first-degree relatives at high autoantibody-inferred risk.
(DOCX)

S1 Table Antibody Table for TT-TRFIA. (DOCX)

S1 File Outprint of the parameters for the adaptation of the TT-TFIA to the Autodelfia 1235 automated instrument (Perkin-Elmer). (DOCX)

S2 File List of the current members of the Belgian Diabetes Registry who participated in the recruitment of relatives and the handling of samples. (DOCX)

S3 File Supporting Information Excel file (XLS)

## Abbreviations

TT-TRFIA: trefoil-type time-resolved fluorescence immunoassay
PI:C: proinsulin:C-peptide ratio
IA-2A: insulinoma-associated protein 2 autoantibody
ZnT8A: zinc transporter 8 autoantibody
PI: proinsulin
OGTT: oral glucose tolerance test
AUC: area under the curve
HbA1c: glycated hemoglobin
IAA: insulin autoantibody
GADA: glutamate decarboxylase autoantibody
ECLIA: electrochemiluminescence immunoassay
AIC: Akaike Information Criterion
ISI: insulin sensitivity index
BMI: body mass index
