# Correction: Prediction of Impending Type 1 Diabetes through Automated Dual-Label Measurement of Proinsulin:C-Peptide Ratio

**DOI:** 10.1371/journal.pone.0179108

**Published:** 2017-06-01

**Authors:** Annelien Van Dalem, Simke Demeester, Eric V. Balti, Bart Keymeulen, Pieter Gillard, Bruno Lapauw, Christophe De Block, Pascale Abrams, Eric Weber, Ilse Vermeulen, Pieter De Pauw, Daniël Pipeleers, Ilse Weets, Frans K. Gorus, Belgian Diabetes Registry

The following information is missing from the Funding section: This study was supported by the Scientific Fund Willy Gepts from the Universitair Ziekenhuis Brussel—UZ Brussel (Grant No WFWG 177).
