# Supplementary material for: Prediction of Impending Type 1 Diabetes through Automated Dual-Label Measurement of Proinsulin:C-Peptide Ratio
Source: PLoS One. 2016 Dec 1;11(12):e0166702. doi: 10.1371/journal.pone.0166702 (PMC5131964; doi:10.1371/journal.pone.0166702)
Supplement: S1 Table — (DOCX) [file pone.0166702.s003.docx]

**S1 Table.**

| **Peptide/protein target** | **Antigen sequence (if known)**  P01308-1* | **Name of antibody** | **Manufacturer, catalog #, and/or name of individual providing the antibody** | **Species raised in; monoclonal or polyclonal** | **Dilution used** |
| --- | --- | --- | --- | --- | --- |
| C-peptide | AA 84-87 | Monoclonal Mouse anti Human C-peptide, Clone PEP-001 | DAKO, Glostrup, Denmark catalog nr OA01313 | Mouse; monoclonal | N/A |
| C-peptide | AA 57-67 | Monoclonal Mouse anti Human C-peptide, CPT-3F11 | DAKO, Glostrup, Denmark catalog nr O958313 | Mouse; monoclonal | 485µg/L |
| Proinsulin | AA 49-52 | Monoclonal Mouse anti Human insulin, HUI-001 | Dr. Pass, Novo Nordisk, Bagsværd, Denmark | Mouse; monoclonal | 35µg/L |

*www.uniprot.org, accessed on June 13^th^, 2016
