# Supplementary material for: Prediction of Impending Type 1 Diabetes through Automated Dual-Label Measurement of Proinsulin:C-Peptide Ratio
Source: PLoS One. 2016 Dec 1;11(12):e0166702. doi: 10.1371/journal.pone.0166702 (PMC5131964; doi:10.1371/journal.pone.0166702)
Supplement: S1 File — (DOCX) [file pone.0166702.s004.docx]

**S1 File.**

The trefoil-type time resolved fluorescence immunoassay for simultaneous measurement of proinsulin and C-peptide in human K-EDTA plasma supplemented with aprotinine [17] was adapted to the Autodelfia 1235 automated analyzer (Perkin-Elmer, Massachusetts, USA). A 96-well microtiterplate (Perkin-Elmer, Massachusetts, USA) was used, coated with PEP-001 antibody. The capture antibody (0.5 mg PEP-001) was dissolved in 15 mL 5 x Antibody coating buffer (Immunochemistry Technologies LLC, USA) and 60 mL MilliQ water. This solution was mixed during 15 minutes on the rolling mixture and was pipetted (180 µL/well) into 96-well microtiterplates (Nunc Maxisorp Yellow). The plates were covered with adhesive polyester film and incubated overnight (shaking). After 4 washings, they were incubated overnight with 300µL/well of Neptune Block Blocking buffer (Immunochemistry Technologies LLC, USA). After aspiration of this solution, the plates were dried and kept at 4°C. They were stable for at least two months. At day 1, the program AUTOSIM.ASS file was started. Calibrators [17], quality control samples and the participants’ plasma (25µL) were pipetted on the PEP-001-coated plate in duplo according to the pipetting scheme. First incubation buffer (40 mmol/L sodium phosphate, 30 g/L purified BSA (Probumin; Millipore), 1 g/L bovine gamma globulin (Sigma–Aldrich), 60 g/L NaCl, 0.5 g/L NaN3, and 1 mL/L Tween 20 (Merck), pH 7.4 at 20 °C) containing 5g/dL fetal bovine serum (100µL) was added to each well and after 2h incubation and shaking, the plate was washed 6 times. All washings were performed with a DELFIA 1296 Platewasher (Perkin-Elmer) filled with a 40-mL/L aqueous dilution of the Wash Concentrate (Perkin-Elmer). After adding 135 µL assay buffer (50 mmol/L Tris-HCl, pH 7.75 at 20 °C, containing 9 g/L NaCl, 0.5 g/L NaN3, 0.5 g/L bovine gamma globulin, 5 g/L purified BSA, 7.44 mg/L disodium EDTA, and 0.1 mL/L Tween 20) containing 485µg/L Eu-CPT-3F11 and 35µg/L BIO-HUI per well, the plate was unloaded, covered and incubated overnight at room temperature under continuous horizontal shaking (50% of maximum speed on a DELFIA 1296 Plateshaker). At day 2, the program SIMULCPE.ASS was started and after 6 washings, 135 µL assay buffer containing 250 µg/L Tb^3+^ - labeled streptavidin was added per well. The plate was incubated under continuous shaking for 1h and after another 6 washing steps Enhancement solution (Perkin-Elmer) (200µL/well) was added. After 5 min of shaking, the Eu^3+^ time-resolved fluorescence was measured to determine the total C-peptide content (in pmol/L). Finally, the SIMULPRO.ASS program was started and 50 µL Enhancer solution (Perkin-Elmer) was added per well. The Tb^3+^ time-resolved fluorescence was measured after 5 min of shaking to measure the proinsulin content (in pmol/L). We then used the smoothed spline algorithm of the Multicalc™ 120 software (version 2.6; PerkinElmer) for regression analysis and to calculate the results for proinsulin and total C-peptide. The concentration of true C-peptide was calculated by subtracting the proinsulin concentration (including the partially converted des and split forms) from the measured concentration of total C-peptide; the concentration of true C-peptide was then used to calculate PI:C as a percentage: 100 x [pmol/L proinsulin] / [pmol/L C-peptide].

1. **AUTOSIM.ASS**

# AUTOSIM.ASS

# *************************************************

# Cassette types and volumes (µl) in reag. Bottles

# in 1 plate and 4 plate kits.

# variables: cassType, Buff1Vol, TracerVol, AntibodyVol, Buff2Vol

# call DspDefs1 2 50000 1500 2500 50000

# call DspDefs4 4 100000 2500 2500 100000

DATA DSPDATA

# **************************************************

BEFORENEXT LOAD SAMPDIL pipetting samples

LOAD SAMPLER

#define wellVol 100 pipetting 100 µL first incubation buffer

DISP PIPBUFB1

SHAKE 7200 600 2h incubation sample in first incubation buffer

WASH WSH6 wash plate

BEFORENEXT DISP DILPRO4

#define wellVol 135 pipetting assay buffer with mAbs

DISP PIPDIL

#inc dilBt1

SHAKE 10 5

#define plateType2

COUNT CNT1

MOVETO SHAKE

/

1. **SIMULCPE.ASS**

# SIMULCPEP.ASS

# *************************************************

# Cassette types and volumes (µl) in reag. Bottles

# in 1 plate and 4 plate kits.

# variables: cassType, Buff1Vol, TracerVol, AntibodyVol, Buff2Vol

# call DspDefs1 2 50000 1500 2500 50000

# call DspDefs4 4 100000 2500 2500 100000

DATA DSPDATA

# **************************************************

WASH WSH6

BEFORENEXT DISP DILPRO3

#define wellVol 135 pipetting assay buffer with Tb-streptavidin

DISP PIPDIL

#inc dilBt1

SHAKE 3600 300 1h incubation with Tb-streptavidin

WASH WSH6

ENHDISP ESD1 pipetting 200 µL Enhancement solution

SHAKE 300 60 5min incubation with Enhancement solution

# define plateType2

COUNT CNT1 measuring “total C-peptide” in pmol/L

MOVETO SHAKE

/

1. **SIMULPRO.ASS**

#MEAS_TB.ASS

# *************************************************

# Cassette types and volumes (µl) in reag. Bottles

# in 1 plate and 4 plate kits.

# variables: cassType, Buff1Vol, TracerVol, AntibodyVol, Buff2Vol

# call DspDefs1 1 50000 0 0 0

# call DspDefs4 3 0 0 0 0

DATA DSPDATA

# **************************************************

#define wellVol 50 pipetting 50µL Enhancer on top of

DISP PIPBUFT1 Enhancement solution

SHAKE 300 10 5min incubation with Enhancer

#define plateType 0

COUNT CNTTB measuring “Proinsulin” in pmol/L

MOVETO SHAKE
