# Supplementary material for: Prediction of Impending Type 1 Diabetes through Automated Dual-Label Measurement of Proinsulin:C-Peptide Ratio
Source: PLoS One. 2016 Dec 1;11(12):e0166702. doi: 10.1371/journal.pone.0166702 (PMC5131964; doi:10.1371/journal.pone.0166702)
Supplement: S2 File — (DOCX) [file pone.0166702.s005.docx]

**S2 File**

Abrams P, St Augustinus, Wilrijk; Algoet C, St Elisabethziekenhuis, Herentals; Annaert M, AZ Diest, Diest; Arnouts P, AZ St Jozef, Turnhout; Ayoubi S, Clinique St Jean, Bruxelles; Bachy A, Clinique Notre Dame, Charleroi; Ballaux D, AZ Nikolaas, Sint-Niklaas; Baltieri D, Hôpital de Jolimont, La Louvière; Bataille G, CH du Grand Hornu-Frameries, Hornu; Beckers V, CH St Joseph, Liège; Beckers D, UCL Mt. Godinne, Yvoir; Becq H, St Augustinus, Wilrijk; Beirinckx J, St Jozefskliniek, Izegem; Beirinckx A, AZ St Lucas, Assebroek; Bellavia S, CHR de Huy, Huy; Benhalima K, UZ Leuven Gasthuisberg, Leuven; Bex M, UZ Leuven Gasthuisberg, Leuven; Bodson A, Polyclinique du Mambourg, Charleroi; Bollaerts K, AZ St Maarten, Mechelen; Bosly F, Clinique St Joseph, Arlon; Bouquegneau MS, CHR Clinique St Joseph, Mons; Bourguignon JP, CHU Sart Tilman, Liège; Bravenboer B, UZ Brussel, Brussel; Brochier S, IFAC Clinique St-Therese, Bastogne; Carlier A, AZ Maria Middelares, Gent; Casteels K, UZ Leuven Gasthuisberg, Leuven; Cavatorta E, CHU Charleroi, Charleroi; Chivu O, CH St Joseph, Liège; Claessens A, Clinique St Joseph, Arlon; Claeys L, AZ St Jozef, Malle; Cnop M, Hôpital Erasme, Bruxelles; Coeckelberghs M, Kinderziekenhuis Paola, Antwerpen; Col V, Clinique St Pierre, Ottignies; Colson A, Clinique St Pierre, Ottignies; Coolens JL, Jessaziekenhuis - Campus Salvator, Hasselt; Coremans P, AZ Nikolaas, Sint-Niklaas; Corvilain B, Hôpital Erasme, Bruxelles; Crenier L, Hôpital Erasme, Bruxelles; Daems T, ZNA Stuivenberg, Antwerpen; Damoiseaux P, CH de Dinant, Dinant; Daoudi N, CHU Charleroi, Charleroi; Daper C, CHU Tivoli, La Louvière; Daubresse C, CH St Joseph, Liège; Daubresse JC, Hôpital Civil Marie Curie, Lodelinsart; De Block C, UZA, Edegem; De Feyter I, AZ Jan Palfijn, Gent; De Grande E, St Andriesziekenhuis, Tielt; De Paepe L, ZNA Stuivenberg, Antwerpen; De Schepper J, UZ Brussel, Brussel; De Schynkel K, AZ Maria Middelares, Gent; De Waele K, UZ Gent, Gent; De Winter P, Heilige Familie, Rumst; Decerf JA, CH de l'Ardenne, Libramont; Declercq E, AZ Alma, Sijsele-Damme; Decochez K, AZ Jan Portaels, Vilvoorde; Decraene P, Imeldaziekenhuis, Bonheiden; Defoer F, Militair hospitaal, Brussel; Degrande E, St Andriesziekenhuis, Tielt; Demuynck S, AZ Heilige Familie, Reet; Den Brinker, UZA, Edegem; Depoorter S, AZ St Jan, Brugge; Derdelinckx L, Clinique Saint Luc, Bouge-Namur; Deweer S, St Elisabethziekenhuis, Zottegem; Dooms L, private, Bree; Dorchy H, HUDERF, Bruxelles; Dotremont H, Kalmthout; Driessens S, AZ KLINA, Brasschaat; Dumasy V, Clinique Louis Caty, Baudour; Duvivier E, Clinique Reine Fabiola, Montignies-sur-Sambre; Duyck F, AZ Delta, Roeselare; Dysseleer A, CH de l'Ardenne, Libramont; Eeckhout B, AZ St Dimpna, Geel; Eenkhoorn V, St Josefkliniek, Bornem; Emsens L, AZ OLV Ter Linden, Knokke-Heist; Engelen W, AZ Stuivenberg, Antwerpen; Ers V, Clinique St Joseph, Arlon; Eykens A, Private, Herentals; Favere N, St Elisabethziekenhuis, Zottegem; Féry F, Hôpital Erasme, Bruxelles; Fils R, Private, Blankenberge; France A, UZA, Edegem; Garmyn K, Private, Lier; Gérard J, CH St Joseph, Liège; Gerniers S, Private, Sint-Denijs-Westrem; Geronooz I, CHR Clinique St Joseph, Liège; Geyskens L, AZ St Jozef, Turnhout; Ghys C, UZ Brussel, Brussel; Gies I, UZ Brussel, Brussel; Gillard P, UZ Leuven, Leuven; Godon E, CHR de Huy, Huy; Gorus F, Brussels Free University-VUB, Brussels; Grabczan L, CHU St Pierre, Bruxelles; Guiot J, Hôpital du Bois de l' Abbaye, Seraing; Haemers S, AZ St Lucas, Gent; Haumont S, Hôpital Erasme, Bruxelles; Herbaut C, CHU Brugmann, Bruxelles; Heyns E, AZ Groeninge Campus OLV, Kortrijk; Hilbrands R, UZ Brussel, Brussel; Huard A, Hôpital de Braine l'Alleud, Braine l'Alleud; Hubermont G, Clinique Princesse Paola, Marche; Huysman F, AZ St Lucas, Gent; Jandrain B, CHU Sart Tilman, Liège; Joosen P, AZ St Jozef, Turnhout; Jopart P, Hôpital de Jolimont, Haine-StPaul; Jousten E, CHR de la Citadelle, Liège; Karmali R, CHU Brugmann, Bruxelles; Keymeulen K, UZ Brussel, Brussel; Kleynen P, CHU Saint-Pierre, Bruxelles; Kockaerts Y, ZOL Campus A. Dumont, Genk; Krzentowski G, Polyclinique de la Madeleine, Jumet; Laga K, St Franciscus Ziekenhuis, Heusden- Zolder; Lamberigts G, AZ St Jan, Brugge; Lambrecht E, AZ Waasland, Sint Niklaas; Lapauw B, UZ Gent, Gent; Lebrethon MC, CHR de la Citadelle, Liège; Lemay P, AZ SintJozef, Turnhout; Lemy C, CHU Charleroi, Charleroi; Leus J, AZ Maria Middelares, Gent; Lienart F, CHU Tivoli, La Louvière; Lim TT, Heilig Hart Ziekenhuis, Mol; Litvine C, CHR Clinique St Joseph, Mons; Logghe K, H Hartziekenhuis, Roeselare; Louis J, Clinique Notre Dame, Charleroi; Lowyck I, ZOL Campus A. Dumont, Genk; Lysy Ph, Clinique Univ St Luc, Bruxelles; Maes M, Clinique Univ St Luc, Bruxelles; Maes T, Imeldaziekenhuis, Bonheiden; Marchal M, CHU Tivoli, La Louvière; Maris E, AZ Nikolaas, Sint Niklaas; Martens M, AZ Sint-Jozef, Turnhout; Massa G, Virga Jesse Ziekenhuis, Hasselt; Mathieu C, UZ Leuven Gasthuisberg, Leuven; Maus Y, Clinique Notre Dame, Charleroi; Mekahli F, Clinique St Jean, Bruxelles; Mekeirele K, OLV van Lourdes, Waregem; Mertens A, Regionaal Ziekenhuis Sint Trudo, Sint-Truiden; Messaaoui A, HUDERF, Bruxelles; Monballyu J, AZ St Jozef, Malle; Moorkens G, UZA, Edegem; Mortelmans K, Regionaal Ziekenhuis Heilig Hart, Leuven; Mouraux T, UCL Mt Godine, Yvoir; Mullens A, Virga Jesse Ziekenhuis, Hasselt; Naudts K, St Elisabethziekenhuis, Zottegem; Nemery A, CHU Ambroise Paré, Mons; Neven I, Clinique Notre Dame, Hermalle /s Argenteau; Neven S, Regionaal Ziekenhuis Sint Trudo, Sint Truiden; Nicolaij D, AZ Groeninge Campus OLV, Kortrijk; Nobels F, OLV Ziekenhuis, Aalst; Nollet A, Regionaal Ziekenhuis Jan Yperman, Ieper; Ooms V, AZ KLINA, Brasschaat; Oriot P, CH de Mouscron, Mouscron; Paciorkowski F, Montigny-le-Tilleul; Paquot N, CH St Joseph, Liège; Paris I, Clinique Notre Dame, Charleroi; Paulussen J, St Elisabethziekenhuis, Herentals; Peeters G, Jan Palfijnziekenhuis, Merksem; Peiffer F, UZA, Edegem; Pelckmans C, AZ St Maarten Campus Duffel, Duffel; Pen J, UZ Brussel, Brussel; Philips JC, CHU Sart Tilman, Liège; Pieron M, CH Peltzer-La Tourelle, Verviers; Pipeleers, Brussels Free University-VUB; Ponchon M, Clinique St Jean, Bruxelles; Poschet K, St Vincentiusziekenhuis, Antwerpen; Rademecker R, CHU Sart Tilman, Liège; Remacle B, Clinique de l'Espérance, Montegnée; Remy C, CHR de la Citadelle, Liège; Renneboog B, CH Tubize-Nivelles, Nivelles; Righes C, CHR de Huy, Huy; Ruige J, AZ Sint-Nikolaas, Sint-Niklaas; Robbrecht S, AZ St Blasius, Dendermonde; Rocour-Brumioul D, CHR de la Citadelle, Liège; Scarniere D, Hôpital St 9 Joseph, Gilly; Scheen A, CHU Sart Tilman, Liège; Schils E, CHU Tivoli, La Louvière; Schoemaker I, Heilig Hartziekenhuis, Lier; Selvais P, Maulde (Tournai); Seret N, CH St Joseph, Liège; Slap F, St Augustinus, Wilrijk; Spijker T, AZ Vesalius, Tongeren; Spincemaille K, H Hartziekenhuis, Roeselare; Stassen-Joly MP, Clinique Andre Renard, Herstal; Strivay M, CHR de la Citadelle, Liège; Taelman P, AZ Maria Middelares, Gent; Taes Y, AZ St Jan, Brugge; Tenoutasse S, HUDERF, Bruxelles; Thielen V, CHR de la Citadelle, Liège; Tits J, ZOL Campus A. Dumont, Genk; Triches K, Hôpital du Bois de l' Abbaye, Seraing; Tshibuabua G, CH de la Basse Sambre, Sambreville; T'sjoen G, UZ Gent, Gent; Tuyttens C, AZ St Lucas, Gent; Unger J, CH Tubize-Nivelles, Nivelles, Unuane D, UZ Brussel, Brussel; Van Acker K, AZ Heilige Familie, Reet; Van Aken E, AZ Diest, Diest; Van Aken S, UZ Gent, Gent; Vanbesien J, UZ Brussel, Brussel; Van Crombrugge P, OLV Ziekenhuis, Aalst; Van de Poel G, UCL Mt. Godinne, Yvoir; Van Den Bruel A, AZ St Jan, Brugge; Van Den Driessche A, St Josefkliniek, Bornem; Van Doninck N, AZ Nikolaas, Sint-Niklaas; Van Doorn J, H Hartziekenhuis, Lier; Van Gaal L, UZA, Edegem; Van Helvoirt M, Private, De Haan; Van Imschoot S, AZ St Jan, Brugge; Van Parys C, RHMS Site Peruwelz, Peruwelz; Van Pottelbergh I, OLV Ziekenhuis, Aalst; Van Poucke K, AZ Nikolaas, SintNiklaas; Van Rooy P, ZNA Middelheim, Antwerpen; Van Winghem C, OLV Middelares, Deurne; Vande Mergel X, CH Tubize-Nivelles, Nivelles; Vandecauter H, AZ Alma, Sijsele-Damme; Vandemeulebroucke E, AZ Jan Portaels, Vilvoorde; Vandenbon C, AZ Damiaan - Campus H. Hart, Oostende; Vandenbroeck P, St Jozefskliniek, Izegem; Vandenbroucke M, AZ St Maarten Campus Mechelen, Mechelen; Vandenbussche E, St Elisabethziekenhuis, Herentals; Vandergheynst A, Hôpital Erasme, Bruxelles; Vanderijst JF, Clinique St Pierre, Ottignies; Vanderijst M, Private, Wavre; Vanderschueren B, UZ Leuven, Leuven; Vanderstappen H, St Franciscus Ziekenhuis, HeusdenZolder; Vandewalle C, ZNA Middelheim, Antwerpen; Van Durme Y, AZ Sint-Augustinus, Veurne; Vanfleteren E, St Jozefskliniek, Izegem; Vanhaverbeke G, AZ Groeninge Campus OLV, Kortrijk; Vanneste S, AZ St Jozef, Malle; Vanuytsel J, AZ Zusters van Barmhartigheid, Ronse; Verbiest R, AZ St Dimpna, Geel; Vercammen C, Imeldaziekenhuis, Bonheiden; Verhaegen A, Jan Palfijnziekenhuis, Merksem; Verhaert G, AZ Monica, Antwerpen; Verjans V, AZ St Jozef, Turnhout; Verniest R, AZ KLINA, Brasschaat; Verschuere J, AZ Zusters van Barmhartigheid, Ronse; Vets B, Imeldaziekenhuis, Bonheiden; Vieillevoye G, Clinique Notre Dame, Charleroi; Vinck W, St Augustinus, Wilrijk; Vinckx J, Imeldaziekenhuis, Bonheiden; Vinken S, ASZ, Aalst; Warnotte C, Clinique Notre Dame de Grace, Gosselies; Watillon P, Clinique Notre Dame de Grace, Gosselies; Weber E, Clinique St Joseph, Arlon; Weemaes I, Private, Turnhout; Weets I, UZ Brussel, Brussel; Winne L, AZ Damiaan - Campus H. Hart, Oostende; Woestenburg A, AZ St Jozef, Malle
